# Effects of Essential Oils from *Cymbopogon* spp. and *Cinnamomum verum* on Biofilm and Virulence Properties of *Escherichia coli* O157:H7

**DOI:** 10.3390/antibiotics10020113

**Published:** 2021-01-25

**Authors:** Raffaella Scotti, Annarita Stringaro, Laura Nicolini, Miriam Zanellato, Priscilla Boccia, Filippo Maggi, Roberta Gabbianelli

**Affiliations:** 1 Biological Service, Italian National Institute of Health, 00161 Rome, Italy; raffaella.scotti@iss.it (R.S.); laura.nicolini@iss.it (L.N.); roberta.gabbianelli@iss.it (R.G.); 2 National Center for Drug Research and Evaluation, Italian National Institute of Health, 00161 Rome, Italy; annarita.stringaro@iss.it; 3 Department of Technological Innovation and Safety of Plants, Product and Anthropic Settlements (DIT), Italian Workers’ Compensation Authority (INAIL), 00143 Rome, Italy; m.zanellato@inail.it (M.Z.); p.boccia@inail.it (P.B.); 4 School of Pharmacy, University of Camerino, 62032 Camerino, Italy

**Keywords:** *Escherichia coli* O157:H7, *Cymbopogon* spp., *Cinnamomum* spp., essential oils, antibacterial activity, biofilm, *Caenorhabditis elegans* model

## Abstract

Every year, the pharmaceutical and food industries produce over 1000 tons of essential oils (EOs) exploitable in different fields as the development of eco-friendly and safe antimicrobial inhibitors. In this work we investigated the potential of some EOs, namely *Cinnamomum verum*, *Cymbopogon martini*, *Cymbopogon* *citratus* and *Cymbopogon flexuosus*, on the growth, biofilm formation and gene expression in four strains of enterohemorrhagic *Escherichia coli* O157:H7. All EOs were analyzed by gas chromatography-mass spectrometry (GC-MS). The antimicrobial activity was performed by using dilutions of EOs ranging from 0.001 to 1.2% (*v*/*v*). Subinhibitory doses were used for biofilm inhibition assay. The expression profiles were obtained by RT-PCR. *E. coli* O157:H7 virulence was evaluated in vivo in the nematode *Caenorhabditis elegans*. All EOs showed minimal inhibitory concentrations (MICs) ranging from 0.0075 to 0.3% (*v*/*v*). *Cinnamomum verum* bark EO had the best activity (MIC of 0.0075% (*v*/*v*) in all strains) while the *C. verum* leaf EO had an intermediate efficacy with MIC of 0.175% (*v*/*v*) in almost all strains. The *Cymbopogon* spp. showed the more variable MICs (ranging from 0.075 to 0.3% (*v*/*v*)) depending on the strain used. Transcriptional analysis showed that *C. martini* EO repressed several genes involved in biofilm formation, virulence, zinc homeostasis and encoding some membrane proteins. All EOs affected zinc homeostasis, reducing *ykg*M and *zin*T expression, and reduced the ability of *E. coli* O157:H7 to infect the nematode *C. elegans*. In conclusion, we demonstrated that these EOs, affecting *E. coli* O157:H7 infectivity, have a great potential to be used against infections caused by microorganisms.

## 1. Introduction

Bacterial infections are becoming a serious healthcare challenge because of the increased dissemination of multi-drug resistant bacteria. In the European Union multidrug resistant infections are responsible for approximately 25,000 patient deaths per year [1]. This increasing resistance of microorganisms to conventional drugs has induced scientists to search for novel substances with antimicrobial activity and with possible minor side-effects. The persistence and the resistance of bacteria to disinfection are often associated with bacterial ability of aggregating to form a biofilm, a complex multicellular community of microorganisms. Biofilm is the predominant lifestyle of bacteria in all environments [2], and is more resistant to antibiotics and disinfectants [3,4].

Enterohemorrhagic *Escherichia coli* O157:H7 (EHEC) is a human pathogen, belonging to the attaching and effacing (A/E) *E. coli* group. It possesses virulence factors essential for adhesion to intestinal epithelial cells (attachment) and responsible for the destruction of the brush border of microvilli (effacement) [5]. This pathogen can cause bloody diarrhea hemorrhagic colitis and approximately four percent of the cases develop hemolytic uremic syndrome (HUS). The use of antibiotics in EHEC infections should be avoided because they induce the SOS response and activate prophages with the release of Shiga toxins [6]. The ability of *E. coli* O157:H7 to adhere and form biofilm on different surfaces, and the absence of effective therapy against EHEC infections have led to developing new antimicrobial agents. In the last years, there has been an increased interest in the study of natural products as possible therapeutic agents, with a particular attention to essential oils (EOs), chemical mixtures produced by the so-called aromatic plants that are known to be active against a wide variety of microorganisms [7]. EOs are complex hydrophobic and volatile liquids containing multiple low molecular weight compounds, including especially monoterpenoids and sesquiterpenoids, but also phenylpropanoids and aliphatic compounds [8]. EOs contain also minor constituents that can inhibit the growth of bacteria and have a synergistic or additive activity to that of major EO components [9,10].

The EOs from cinnamon (*Cinnamomum verum* J. Presl), lemongrass (*Cymbopogon citratus* DC. Stapf and *C. flexuosus* (Nees ex Steud.) W. Watson) and palmarosa (*C. martini* (Roxb.) W. Watson) are produced in high quantity by the industry (between 50 and 100 tons/year) and are potentially exploitable in different fields. Notably, their antimicrobial activities are widely recognized and documented by the scientific community [11,12,13,14]. Indeed, EOs have previously shown a great potential against gastrointestinal and other pathogens [15,16,17]. Although plant extracts and EOs have been shown to have anti-*E. coli* O157:H7 activity [9,16,18], comparative studies of the effects of EOs in *E. coli* EDL933 reference strain and in clinical human isolated from patients are limited. Here we focused on the five most promising EOs, chosen from a panel of ten EOs used in a first screening to test the inhibition of biofilm in different *E. coli* strains.

The aim of this study was to investigate the antibacterial and antibiofilm activities of five EOs, namely those obtained from bark and leaves of *C. verum* and from *C. citratus*, *C. flexuosu*s and *C. martini*, on several *E. coli* O157:H7 strains and to explore the possible targets of their action. Thus, we analyzed the expression of genes related to biofilm formation and pathogenesis in *E. coli*. Since gene expression studies conducted on *E. coli* cells treated with *C. martini* are not known, we initially investigated the transcription levels of genes involved in motility (*csg*A, *fli*A and *fim*A), virulence (*stx*2 and *eha*A), synthesis of membrane proteins (*agn*43, *omp*A and *pga*A) and zinc homeostasis (*ykg*M and *zin*T) in strains grown in the presence of *C. martini*. Finally, we also analyzed the transcription levels of *ykg*M and z*in*T in cells treated with the other EOs. Furthermore, an in vivo *Caenorhabditis elegans* model [19,20] was used to study the effects of the EOs on the infection capacity of *E. coli* O157: H7.

## 2. Results and Discussion

### 2.1. Chemical Components of Essential Oils

The chemical compositions of the five EOs, as determined by GC-MS analysis, are reported in Supplementary Materials (Figure S1) and the main components are illustrated in Table 1. The analysis of EO chemical constituents (Supplementary Materials Table S1) showed that the main components were oxygenated monoterpenes and phenylpropanoids for *Cymbopogon* spp. and *C. verum* EOs, respectively. The main compounds of lemongrass EOs, which are represented by the species *C. citratus* and *C. flexuosus*, were the monoterpene aldehydes neral (32.0% and 30%, respectively) and geranial (48.2% and 41.5%, respectively). Their mixture is commonly named citral [21]. *C. martini* EO was characterized by the monoterpene alcohol geraniol (82.2%) and its ester geranyl acetate (11.1%). The *C. verum* bark and leaf EO compositions were dominated by the phenylpropanoids (*E*)-cinnamaldehyde (85.4%) and eugenol (83.5%), respectively. Geraniol and citral were reported as active antimicrobial agents due to their functional groups and to their affinity for microbial membranes, that they traverse interacting with vital metabolic enzymes [8,22]. It was reported that (*E*)-cinnamaldehyde is one of the most potent natural antibacterial substances [23,24]. Its action, as well as that of eugenol, is conferred by free aldehyde and hydroxyl groups [25,26]. These are highly reactive and form hydrogen bonds with the active site of target enzymes, inactivating them [27,28], and with the cell membrane, damaging it [8,29]. Although percentages of tested EOs differed in some degree from those previously reported, the main components were the same [30,31,32,33,34,35]. It is known that the different EO extraction methods, cultivation geographical region and climatic conditions can be responsible for these variations [1,31].

### 2.2. Antibacterial and Antibiofilm Activity of EOs

EOs exhibited remarkable *in vitro* antibacterial activity against all strains tested, as indicated in the results of the MIC determination (Table 2). The planktonic growth curves (data not shown) of examined strains indicated MIC values varying from 0.075% to 0.3% depending on EO and strain used. *C. verum* bark EO had the strongest inhibitory effect, showing a value of MIC of 0.0075% in all strains. The ED597 was more sensitive to the assayed EOs than the other strains, while the K12 was poorly sensitive to the EOs treatment. Minimal bactericidal concentration (MBC) values were above MIC values for most EOs (between 2× MIC and 4× MIC), while for the *C. verum* bark EO they were even 10× MIC. Only the *C. verum* leaf EO, MIC and MBC values were not very different.

As reported in previous papers [6,16,19,30,34,36,37], all EOs used in this study had an inhibitory effect on the planktonic growth of *E. coli* O157:H7, with some differences in strength of action. In addition, we reported that *C. verum* bark EO had a bactericidal effect on all strains used at a very low concentration (between 0.02% and 0.08% *v*/*v*), a finding supported by the work of Sheng et al. [6] where *C. verum* EO was found to be bactericidal on *E. coli* O157:H7 at 0.05% (*v*/*v*). Chloramphenicol and kanamycin, used as reference controls for the tested bacteria, showed a value of MIC of 4 and 8 μg/mL in all strains, respectively (data not shown).

**Figure 1:**
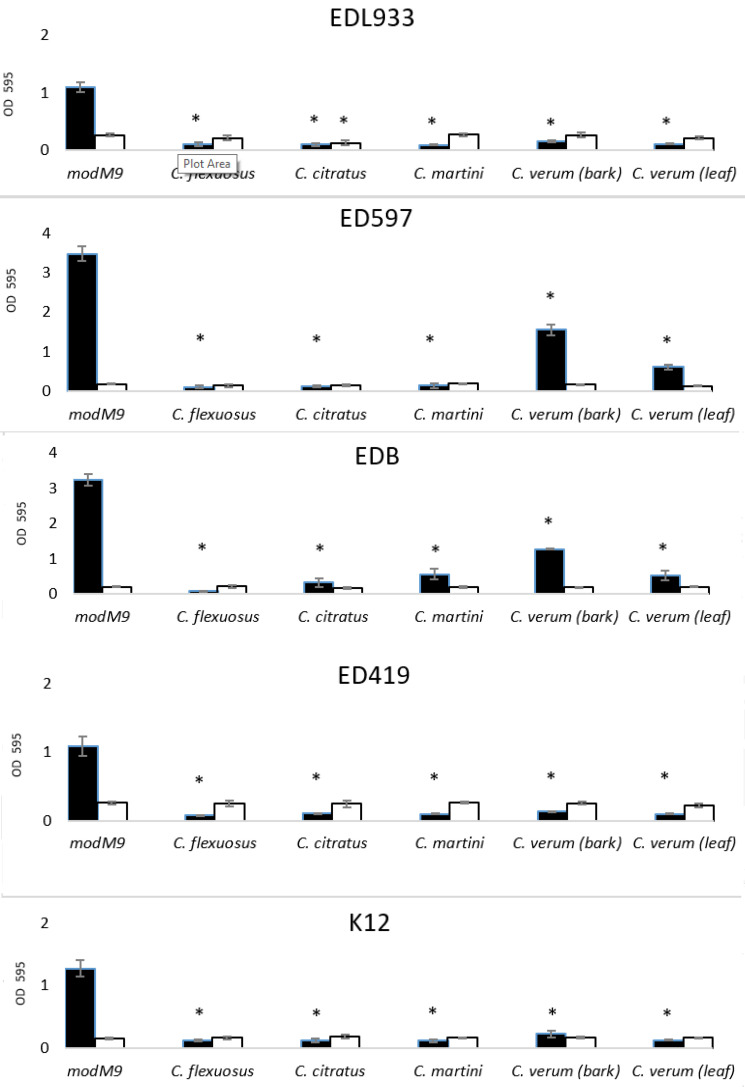
Antibacterial and antibiofilm activity of EOs on *E. coli* strains. Planktonic growth (white bars) and biofilm formation (black bars) were quantified in modM9 and in modM9 in the presence of EOs, after culture of 24 h in 96-well plates, at 28 °C. Data are presented as mean ± SD of absorbance (at 595 nm). * *p* < 0.01.

*E. coli* is a part of gut/intestinal microbiota, with the ability to form biofilm on abiotic and biotic surfaces. To investigate the effect of EOs on the ability of bacteria to form biofilm we used the EOs at the subinhibitory concentration of 0.05% (*v*/*v*) (the cinnamon bark EO at 0.005% (*v*/*v*)). Figure 1 showed that all strains were greatly inhibited in their capacity to form biofilm on polystyrene plates, with a percentage of inhibition between 55% and nearly 100%. *Cymbopogon* spp. EOs had the greatest antibiofilm activity against all strains. On the other hand, *C. verum* bark EO had a more potent bactericidal and antibiofilm effect, since it was used in this study at 10 times lower concentrations. To date, several EOs were reported to have an activity against *E. coli* O157:H7 [31,38]. Kim et al. [19] investigated the effect of 83 EOs at concentration of 0.005% (*v*/*v*) in Luria-Bertani medium on biofilm formation in *E. coli* O157:H7 EDL933 strain, including *C. citratus*, *C. martini* and cinnamon bark EOs from *Cinnamomum cassia* (L.) J. Presl. Our results with *C. verum* bark EO agree with those by Kim et al. [19]; however, with *C. citratus* and *C. martini* EOs we obtained a greater inhibition, probably due to the higher concentration we used. Interestingly, the planktonic growth was not affected at the concentrations used in the biofilm assay except for a lesser extent in the presence of *C. citratus*, suggesting that the inhibition of biofilm was not a consequence of the absence of planktonic growth; however, it was due to the antibiofilm activity of EOs.

### 2.3. Gene Expression Analysis

**Figure 2:**
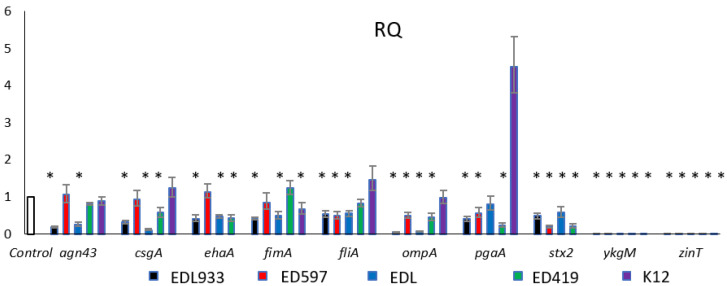
Expression levels of selected genes related to biofilm formation and pathogenesis in *E. coli* strains treated with or without *C. martini* EO at 0.05% (*v*/*v*) concentration. Transcriptional profiles were measured by qRT-PCR. * *p* < 0.01.

In order to understand the molecular mechanisms behind the inhibitory role of EOs in pathogens, some genes related to biofilm formation and pathogenesis of *E. coli* were monitored by quantitative real-time PCR. We studied the genes involved in motility (*csg*A, *fli*A and *fim*A), virulence (*stx*2 and *eha*A), membrane protein synthesis (*agn*43, *omp*A and *pga*A) and zinc homeostasis (*ykg*M and *zin*T) in the presence of *C. martini* EO. Indeed, in the literature, the studies on *C. martini* EO are very limited and, to our knowledge, this is the first gene expression study with this oil. Thus, initially we analyzed gene expression levels of mRNA isolated from cells treated with *C. martini* EO. As shown in Figure 2, the examined genes were mainly downregulated in the pathogen strains. The genes involved in the virulence are missing in the non-pathogen K12 strain. In our control EDL933 strain all genes were downregulated, as well as in EDB strain, in which only the *pga*A gene was not differentially expressed. *pga*A gene belongs to the *pga*ABCD operon which synthesizes the poly-β-1,6-N-acetyl-D-glucosamine, essential for the cellular architecture of the *E. coli* biofilm structure. In particular, this gene codes for the exporter of the PGA protein outside the periplasm space. It is noteworthy that this gene was instead highly expressed in the non-pathogen K12, possibly due to a different role of PGA in biofilm formation on plastic material in *E. coli* O157:H7 and K12 strains [39]. Interestingly, *omp*A and *stx*2 were downregulated in all considered strains.

The OmpA is a predominant antigen in the outer membrane, and it can function as an adhesion factor and invasion and serves as a receptor for several bacteriophages. It is known to increase the biofilm formation [40,41] and Barrios et al. [42] demonstrated that *omp*A mutants formed biofilm of much reduced thickness. Torres et al. [43] described a role as a virulence factor in *E. coli* O157:H7, which utilized OmpA in adhesion to host cells. Our results demonstrated that *C. martini* EO repressed *omp*A expression in all tested pathogen strains grown in the minimal medium M9, indicating that this gene plays a crucial role in the biofilm formation. The *stx*2 gene codes for the Stx2 which is an important member of the Shiga toxins family and is associated with the more severe disease in humans. It is a protein essential to the pathogenesis of *E. coli* O157:H7 because, after binding to its receptor, the toxin–receptor complex is internalized. Stx2 production in the gastrointestinal tract, in conjunction with other virulence factors, induces hemorrhagic colitis and its entry into the circulatory system can lead to HUS [6].

**Figure 3:**
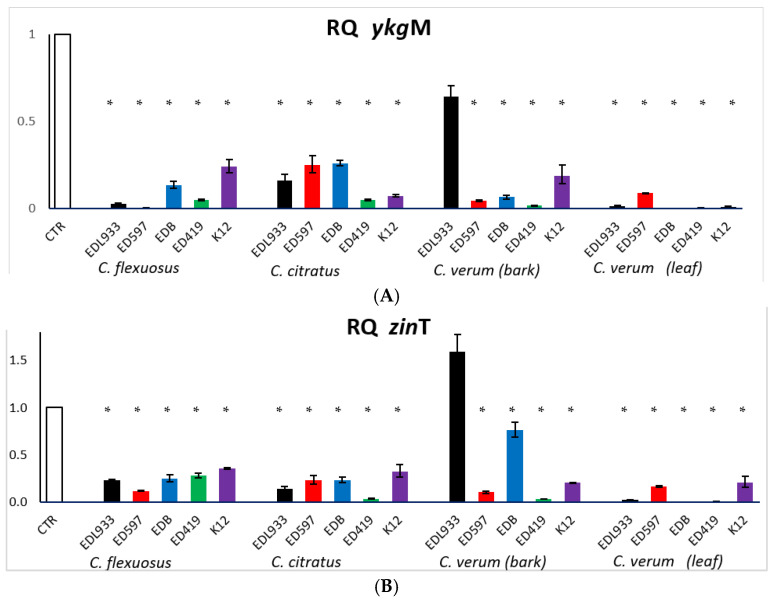
Transcriptional profiles of *ykg*M (**A**) and *zin*T (**B**) in *E. coli* cells treated with or without EOs (CTR), obtained by qRT-PCR. Relative gene expressions represent transcriptional levels after exposure to EOs versus untreated controls. The experiment was performed three times. * *p* < 0.01.

In the literature, many works on the effect of EOs on *stx* repression [6,30,44,45] are reported. Takemasa et al. [46] reported that 20 spices tested, containing eugenol as a main component, were able to reduce Stx production in *E. coli* O157:H7. However, this is the first study that reported the effect of *C. martini* on *stx* expression where, using a sublethal concentration of EO, we obtained inhibition of *stx*2 mRNA expression in all strains examined, except in K12 where the gene is absent. It is intriguing to note how *zin*T and *ykg*M expression was completely repressed in all strains treated with *C. martini*. Due to the relevant role of these two genes in zinc homeostasis and biofilm formation in zinc-deficient conditions [47], to verify if the other EOs also had the same effect, the *ykg*M and *zin*T expression was examined in cells treated with the other EOs (Figure 3).

Only the *C. verum* leaf EO caused an extensive downregulation of the two genes, as observed with *C. martini* EO. The *C. flexuosus*, *C. citratus* and *C. verum* bark EOs affected the *zin*T and *ykg*M expression to a weaker but significant extent and, unexpectedly, we observed that *zin*T gene was induced and *ykg*M expression was repressed in the EDL933 cells treated with the *C. verum* bark EO. These contrasting results will require further investigations. Nevertheless, findings from transcription analysis of *zin*T and *ykg*M genes suggested that the antibiofilm activity of tested EOs on *E. coli* could also be related to the homeostasis of zinc. Moreover, it is known that if the zinc homeostasis is not maintained, a series of effect will take place, including an inhibition of curli formation, bacterial attachment, biofilm formation [47] and Stx toxin production [48].

### 2.4. Scanning Electron Microscope Analysis

We used the scanning electron microscope (SEM) to observe the shape of *E. coli* cells and changes in cell morphology under influence of EOs, analyzing the biofilm of all strains with or without treatment with the tested EOs.

**Figure 4:**
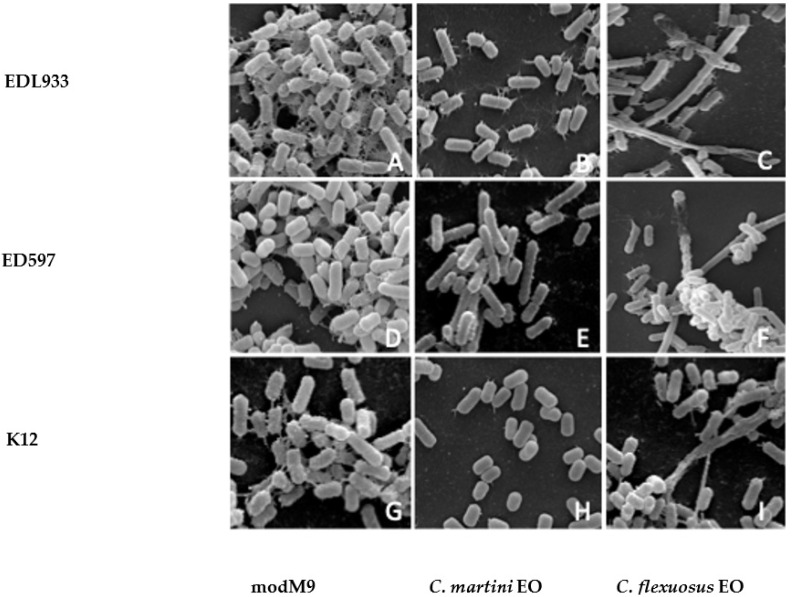
Effect of *C. martini (***B**,**E**,**H***)* and *C. flexuosus* (**C,F**,**I**) EOs on biofilm formation by *E. coli* strains (EDL933 strain A-C, ED597 strain D-F, K12strain G-I). Scanning electron microscope (SEM) was used to examine biofilm cells grown on glass coverslips in modM9 in the presence (**B**,**C**,**E**,**F**,**H**,**I**) or absence (**A**,**D**,**G**) of EOs for 24 h (magnification 5000×).

The results obtained showed that *E. coli* O157:H7 strains had a similar behavior, thus in Figure 4 we reported only EDL933, ED597 and the non-pathogen strain. Although this method of investigation does not allow for quantitative assessments, it was evident from the SEM images that the number of cells was higher in modM9 than in strains treated with EOs. In addition, bacteria grown in minimal medium formed a biofilm composed of intact cells, organized in a dense multilayer structure, embedded in a rich matrix network of polysaccharide material (Figure 4A,D,G). In accordance with the results of inhibition biofilm assay in presence of EOs, the cells exhibited unorganized biofilm cells, scattered and interconnected by a sparse network (Figure 4B,C,E,F,H,I). SEM observations showed considerable morphological alterations in cells treated with *C. flexuosus* EO (Figure 4C,F,I), similar to those observed with *C. citratus* EO (data not shown). In fact, the biofilm of each strain grown in modM9 was composed of rod-shaped cells, smooth, swollen and structured in a multilayer. In the presence of *C. flexuosus* or *C. citratus* EOs, the entire organization of the biofilm was altered, and the single cells appeared with evident morphological changes, becoming very elongated with loss of turgidity. SEM micrographs of biofilm obtained in the presence of *C. martini* and *C. verum* EOs (only data for *C. martini* EO are shown) highlighted sparse micro-colonies and individual cells with fewer and shorter interconnecting meshes between cells, but without evident morphological alterations (Figure 4B,E,H).

### 2.5. EOs Antibacterial Activity in the Nematode Model

**Figure 5:**
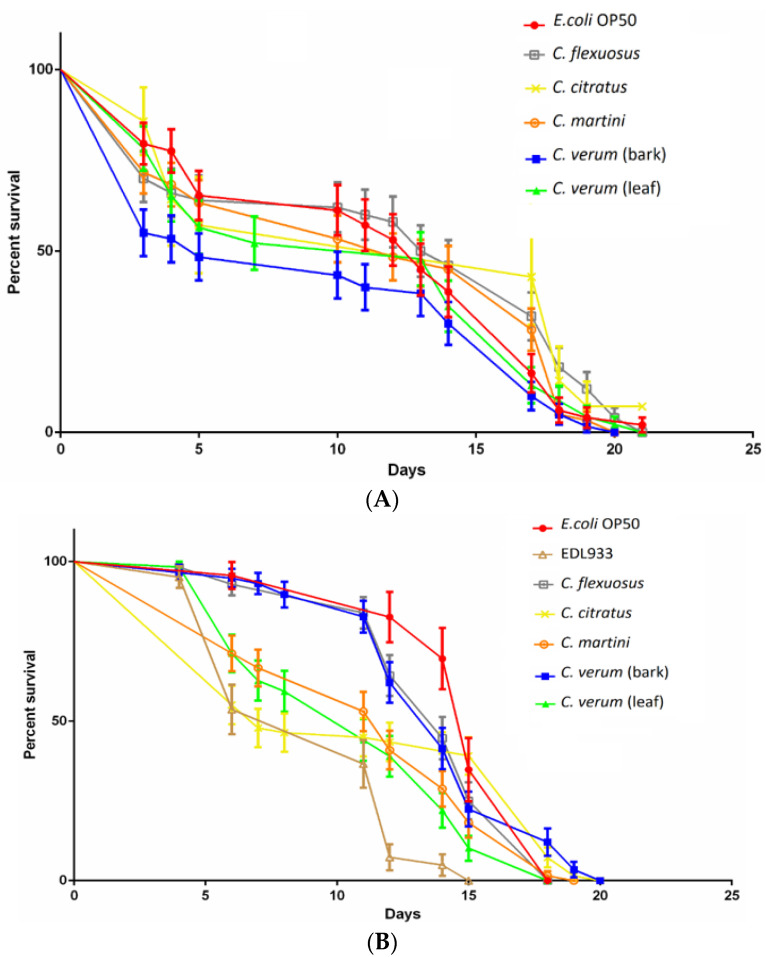
(**A**) Effect of EOs on survival of *C. elegans* infected with *E. coli* OP 50. Nematodes infected with OP 50 without oil were used as control. (**B**) Effect of EOs on the survival of *C. elegans* infected with EDL933. Nematodes infected with *E. coli* OP 50 and EDL933 in the absence of EOs were used as controls. The experiment was performed three times.

Nematode lifespan was monitored in the presence and in absence of the tested EOs to evaluate their potential toxic effects on *C. elegans* vitality. The graph in Figure 5A showed how treatment with the tested EOs did not significantly affect the lifespan of the nematodes, indicating that the EOs did not exert a negative effect, contrary to the report by Kumaran et al. [49] on *C. martini* EO. Subsequently, the nematode lifespan was used as a parameter to evaluate if the treatment with EOs could inhibit the *E. coli* O157:H7 virulence [19,20]. Figure 5B shows how untreated EDL933 significantly reduced (15 days) the lifespan of nematodes compared with the 21 days of treated EDL933 and the negative control (*E. coli* OP50). These results, obtained in the nematode in vivo model, demonstrated for the first time that *C. flexuosus, C. citratus* and *C. martini* EOs, as well as *C. verum* EOs, were able to attenuate the EDL933 virulence. Chou et al. [50] demonstrated that *stx*1 was required for full toxicity of *E. coli* O157:H7 in *C. elegans*. In agreement with previous results of repression of *stx* by EOs [6,19] it was possible that the tested EOs reduced the lifespan of *C. elegans* repressing the expression of *stx*1, with a similar effect demonstrated for *C. verum* EO by Erfan et al. [51]. The study of the expression of *stx*1 in presence of *Cymbopogon* spp. and *C. verum* EOs will be able to provide new insights into the mode of action of these EOs.

## 3. Materials and Methods

### 3.1. Essential Oils

Commercial EOs used in this study were from lemongrass (*C. flexuosus* and *C. citratus*), palmarosa (*C. martini*) and cinnamon bark and leaf (*C. verum*). They were purchased from Naissure Traiding (Neath, Dyfes, UK). EOs were stored at 4 °C and in the dark until use. The EOs quality control for antibacterial activity was tested before the experiments. EOs were streaked onto an LB agar plate and the absence of colonies after the incubation of 24 h at 37 °C confirmed the EOs sterility. To prepare a 10% (*v*/*v*) stock solution of EOs, 50 µL of EO were dissolved in 450 μL of dilution buffer (10% DMSO, 0.5% Tween 80 in PBS) before using.

### 3.2. Gas Chromatography-Mass Spectrometry (GC-MS) Analysis

The chemical compositions of the EOs were analyzed by gas chromatography-mass spectrometry (GC-MS) using an Agilent 6890 N chromatograph coupled with a single quadrupole Agilent 5973 N. The injection was achieved by an autosampler Agilent 7863. The mobile phase was helium (99.999%) whereas the stationary phase was an HP-5MS (30 m × 0.25 mm, 0.1 μm i.d.) capillary column from Agilent. The analytical conditions, including injection, split mode, temperature program, scan mode, as well as the identification and quantification of components, were the same as those reported by Maggi et al. [52] and Ornano et al. [53].

### 3.3. Bacterial Strains

In this study, all in vitro tests were performed using the *E. coli* O157:H7 reference strain EDL933 and three clinical human isolates (ED597, EDB and ED419), belonging to our laboratory collection [54]. As a non-pathogen, *E. coli* K12 reference strain MG1655 was used. The cultures were obtained by diluting with minimal medium modM9 [55] and measuring OD at 595 nm to obtain a reading of 0.05–0.07, giving a standardized inoculum of 1 × 10^6^ CFU/mL. To prepare the modM9 as well as other zinc-free solutions, ultra-pure water produced by a reverse osmosis system characterized by conductivity lower than 0.03 μ_S_/cm was used. Moreover, bacterial culture and all solutions used with modM9 were prepared, incubated using zinc-free materials and controlled as described by Gabbianelli et al. [55].

### 3.4. MIC and MBC Assay

All used strains were grown in the modM9 in the presence of EOs, and their growth in modM9 was used as control. An overnight inoculum was added to modM9 at the final concentration of 1 × 10^6^ CFU/mL in a total volume of 200 μL and inoculated in triplicate in a 96-wells polystyrene plate (Becton Dickinson). For determining the MICs, experiments were performed by the broth microdilution method, adapted from CLSI [56]. Two-fold dilutions of EOs, with additional intermediate dilutions, were made to produce a range of EOs concentrations from 0.018 to 1.2% (*v*/*v*). *C. verum* bark EO was used at final concentrations about 10 times lower than the other EOs, from 0.001% to 0.018% (*v*/*v*). Two standard reference antibiotics, chloramphenicol and kanamycin (32–1 μg/mL), were used as reference controls for the tested bacteria and were obtained by twofold microdilution method. The microplate was incubated at 28 °C with constant agitation for 24 h and optical density monitored at 595 nm every hour in a microplate reader (ELX808, BIO-TEK Instruments). Minimal inhibition concentration (MIC) was determined as the lowest concentration of each EO that completely inhibited the growth of planktonic cells. Subcultures of 100 µL were taken from the clear wells of the microplate and streaked onto agar LB plate, allowing determination of minimal bactericidal concentration (MBC). These values were determined at the lowest concentration of EO where no growth was observed. All tests were performed in triplicate.

### 3.5. Biofilm Formation Assay

A static biofilm formation assay was performed in 96-wells polystyrene plates, as previously reported [57]. Briefly, overnight cultures were inoculated in modM9 in the absence or presence of EOs at an initial concentration of 1 × 10^6^ CFU/mL in a total volume of 200 µL, inoculated in 96-wells microplate and incubated at 28 °C for 24 h. Different subinhibitory concentrations of EOs, ranging from 0.1 to 0.05% (*v*/*v*), were added to cultures. *C. verum* bark EO has been used at a concentration 10 times lower, ranging from 0.01 to 0.005% (*v*/*v*). To quantify total biofilm formation, cell cultures were washed three times with PBS, to remove non-adhered cells, and air dried for 1 h. Biofilm was stained with 0.1% crystal violet for 20 min and rinsed three times with tap H_2_O, extracted with DMSO and absorbance measured at 595 nm. Results are the average of at least nine replicates.

### 3.6. Scanning Electron Microscopy (SEM)

Scanning electron microscopy (SEM) was used to assess the morphological effects on biofilm formed by *E. coli* in presence of EOs at 0.025% (*v*/*v*), the minimal subinhibitory concentration that allowed the biofilm formation on glass coverslips, while showing the damages. Both untreated and treated biofilms were obtained as described in our previous work [57]. Briefly, biofilms formed on coverslips of 12 mm diameter were fixed with 2.5% glutaraldehyde in 0.1 g/L^−1^ sodium cacodylate buffer, pH 7.4 at room temperature for 30 min. The fixed cells were then washed three times with the same buffer and post fixed with 1% osmium tetroxide for three weeks at 4 °C. These samples were washed twice with cacodylate buffer and then dehydrated using a graded alcohol series. After the passage in 100% ethanol, the samples were critical point-dried in CO_2_ (CPD 030 Blazers device, Bal-Tec, Blazers) and gold coated by sputtering (SCD 040 Blazers device, Bal-Tec). The samples were examined with a scanning electron microscope FEI Quanta Inspect FEG, (FEI, Hillsboro, OR, USA).

### 3.7. RNA Isolation and Quantitative Real-Time RT-PCR

To isolate the RNA, the strains were inoculated in 8 mL of modM9, at the initial concentration of 1 × 10^7^ CFU/mL in the presence or absence of EOs, used at a sublethal concentration (0.05% (*v*/*v*) for *C. flexuosus*, *C. citratus*, *C. martini* and *C. verum* leaf EOs and 0.005% (*v*/*v*) for *C. verum* bark EO) with no inhibitory effect on bacterial growth. Cultures were incubated at 28 °C for 24 h with 250 rpm agitation and stabilized with RNA Protect Bacteria Reagent (Qiagen). RNA extraction was performed using Presto mini-RNA Bacteria kit (Geneaid), DNA contamination was eliminated using DNase I (Epicentre) for 20 min at 37 °C and RNA was precipitated with 0.7% isopropanol and 0.3 M sodium acetate. The absence of residual DNA was verified by PCR using specific primers. The quantity and the integrity of RNA were determined using a UV-VIS one-drop micro-volume spectrophotometer (DeNovix-Resnova) at 260 nm. RT-PCR was used to investigate the transcription levels of genes involved in motility (*csg*A, *fli*A and *fim*A), virulence (*stx*2 and *eha*A), synthesis of membrane proteins (*agn*43, *omp*A and *pga*A) and zinc homeostasis (*ykg*M and *zin*T) in *E. coli* treated with or without EO of *C. martini* 0.05% (*v*/*v*). The expression of *ykg*M and *zin*T genes was analyzed also using the other EOs at the concentration of 0.05% (*v*/*v*) except for the *C. verum* bark EO which was used at 0.005% (*v*/*v*). To perform RT-PCR a SYBR green kit (Luna Universal One-Step RT-qPCR, BioLabs) was used. The PCR cycling conditions were as follows: cDNA synthesis at 55 °C for 10 min; denaturation program of 95 °C for 1 min; amplification and quantification repeated for 40 times (95 °C for 10 s and 60 °C for 30 s). The specificity of PCR was determined with melting curve analyses (55° to 95 °C with a heating rate of 0.3 °C/s). Forward and reverse primers (Table 3) for the analyzed genes were designed using Pel Primer software. To determine the efficiency of each primer pair, a series of five ten-fold dilutions were performed, and standard curves were generated. R2 values, or correlation coefficients >0.95 were considered an optimal correlation between values. The 16 s rRNA, a housekeeping gene, was used to normalize the levels of target gene expression detected between treated and untreated strains by measuring the changes in fold expression using the 2-ΔΔCT method [58]. Bacteria without treatments were used as calibrator sample (control sample). All RT-PCR experiments were conducted at least in triplicate.

### 3.8. Caenorhabditis Elegans Killing Assay

The in vivo antibacterial activity of EOs was tested on the *C. elegans* model system. Wild-type nematodes (N2 strain) were cultivated in Petri dishes containing nematode growth media (NGM) agar and a food source consisting of *E. coli* OP50 grown as a superficial monolayer. The nematodes were synchronized to obtain a colony of individuals at the same larval stage. First, 24-wells plates, containing 1 mL of NGM each well were prepared with 100 µM of ampicillin to avoid bacterial contaminations and 40 mM of 5-fluoro-2′-deoxyuridine (FUDR) to obtain sterile nematode was added to NGM agar. In order to evaluate the potentially toxic effects of EOs on *C. elegans* vitality, 50 µL of *E. coli* OP50 liquid culture were seeded into each well and incubated overnight at 37 °C to obtain a bacterial monolayer, with 50 µL of 0.05% (*v*/*v*) of *C. flexuosus*, *C. citratus*, *C. martini* and *C. verum* leaf EOs and 0.005% (*v*/*v*) of *C. verum* bark EO added to each well. Moreover, to assess the effects of EOs on *E. coli* O157:H7 ability to kill the nematode, an inoculum of 1 × 10^6^ CFU/mL of EDL933 strain was grown overnight at 28 °C in the modM9 in the absence or presence of EOs and 50 μL of the treated and untreated inoculate containing EDL933 were added to each well of the dedicated plate. No treatment was applied to the negative control dedicated plate where *E. coli* OP50 was added. After preparing the 24-wells plates as described above, about 40 synchronized L4/young adult nematodes were transferred into plates (four nematodes for well) and scored as dead or alive on a daily basis by gently touching them with a platinum wire. Three independent experiments were conducted (approximately 100 L4/young adult nematodes tested for each treatment).

### 3.9. Statistical Analysis

Experiments were performed in triplicate and repeated for at least three times (*n* = 9). Results were presented as average ± the standard deviations, and statistically significant differences (*p* < 0.01) were determined by Student’s t-test using Excel software (Microsoft Office Excel 2016). *C. elegans* killing assay statistical analysis was performed by long-rank test (Mantel–Cox) [59].

## 4. Conclusions

EOs extracted from aromatic plants of commercial interest possess strong antimicrobial activity and the findings of this study support previous reports on the effect of EOs. The results emerged from this study demonstrated that EOs can inhibit bacterial growth and reduce the ability to form a biofilm. The EOs from *C. verum* and *Cymbopogon* species demonstrated to be effective against several pathogen *E. coli* O157:H7 strains. Particularly, *C. martini* EO interferes with the expression of genes that are directly or indirectly responsible for the formation of biofilm and genes implicated in the pathogenesis. Furthermore, the present study suggests that the activity of also EOs involves the homeostasis of zinc and confirms a possible action on the cellular membrane. In conclusion, we confirm the usefulness of EOs as promising alternative candidates to current antimicrobials in the EHCH infections, mainly due to the SOS response and production of Shiga toxin induced by antibiotics. On the contrary, EOs appear to reduce the expression of *stx*, which is the trigger for the onset of HUS. However, due to the complex action displayed by EOs, further research is needed to elucidate the molecular mechanisms underlying the noteworthy antibacterial activity in order to support their future exploitation as natural antibiofilm agents. The main limitation in the use of EOs as antimicrobial agents is due to their complex composition, which depends on various factors (i.e., extraction method, geographical area of origin etc.) which entails the need for accurate control of the individual batches.

## Figures and Tables

**Table 1:** The main components present in the tested essential oils (Eos) at relative percentages higher than one percent.

EO	Main Components
*Cymbopogon flexuosus*	geranial (41.5%), neral (30.0%), geranyl acetate (6.1%), geraniol (5.5%), caryophyllene oxide (2.8%), γ-cadinene (2.6%)
*Cymbopogon citratus*	geranial (48.2%), neral (32%), geranyl acetate (3.8%), geraniol (3.1%), camphene (1.9%), (*E*)-caryophyllene
*Cymbopogon martini*	geraniol (82.5%), geranyl acetate (11.1%), (*E*)-caryophyllene (2.2%), linalool (2.1%)
*Cinnamomum verum* (bark)	(*E*)-cinnamaldehyde (85.4%), eugenol (7.0%), (*E*)-cinnamyl acetate (4.5%)
*Cinnamomum verum* (leaf)	eugenol (83.5%), benzyl benzoate (2.9%), (*E*)-caryophyllene (2.8%), eugenol acetate (1.9%), α-humulene (1.2%), (E)-cinnamyl acetate (1.2%)

**Table 2:** Minimum inhibitory and bactericidal concentration of EOs against *E. coli* strains. The results shown are in percentage (*v*/*v*) and from three independent experiments.

Strain/EO	*C. flexuosus*	*C. citratus*	*C. martini*	*C. verum* (Bark)	*C. verum* (Leaf)
EDL933	MIC 0.075	MIC 0.075	MIC 0.100	MIC 0.0075	MIC 0.175
	MBC 0.400	MBC 0.200	MBC 0.400	MBC 0.0800	MBC 0.200
ED597	MIC 0.075	MIC 0.075	MIC 0.075	MIC 0.0075	MIC 0.150
	MBC 0.300	MBC 0.200	MBC 0.300	MBC 0.0200	MBC 0.200
EDB	MIC 0.075	MIC 0.200	MIC 0.100	MIC 0.0075	MIC 0.175
	MBC 0.400	MBC 0.400	MBC 0.200	MBC 0.0600	MBC 0.200
ED419	MIC 0.100	MIC 0.100	MIC 0.100	MIC 0.0075	MIC 0.175
	MBC 0.300	MBC 0.200	MBC 0.200	MBC 0.0800	MBC 0.300
K12	MIC 0.150	MIC 0.200	MIC 0.300	MIC 0.0075	MIC 0.175
	MBC 0.400	MBC 0.400	MBC 0.400	MBC 0.0400	MBC 0.300

MIC = minimal inhibitory concentration, MBC = minimal bactericidal concentration.

**Table 3:** Primers used in this study for reverse transcription-quantitative PCR.

Oligo Name	Sequence 5’-3’
*ant*43 F/R	ACAAATGGTCGTCAGGTCGT
*csg*A F/R	CCCGTATACGAGTTGTCAGA
	GCTCAATCGATCTGACCCAA
*eha*A F/R	TTACCAAAGCCAACCTGAGT
	CAGCCGTTTGTAGAAGTGAA
*fim*A F/R	GCAGAGGTGTCATTATATCCC
	CGTTCAGTTAGGACAGGTTC
*fli*A F/R	TTATTCAGGGTTGTTTGCTCA
	GTAAGTTGTAAATGCCGTTCC
*pga*A F/R	GCTGAAGGTGTAATGGATAAAC
	AGGGACTGCGCATTGATTAC
*omp*A F/R	GTTCACGTTCGACAACATCG
	GTTGTAAGCGTCAGAACCGA
*stx*2 F/R	ACAGACCAAGCACTTCACTC
	CGTTCCGGAATGCAAATCAG
*ykg*M F/R	GCGTCATCGTATACACAGGA
	TACTGTGGTGTTCCACGACACC
*zin*T F/R	CCTGTATAGAACGGGTGCGATT
	ACGGCAAACCCTTAACAGA
*16 s* F/R	CTCCATCCCAGTCACTGAG
	CATCCACAGAACTTTCCAGAG
	CCAACATTTCACAACACGAG

## Data Availability

Data available on request.

## Supplementary Materials

The following are available online at https://www.mdpi.com/2079-6382/10/2/113/s1, Figure S1: GC-MS chemical profiles of the five essential oils investigated for the antibiofilm activity, Table S1: Chemical composition (%) of essential oils.
