# Supplementary material for: Effects of Essential Oils from Cymbopogon spp. and Cinnamomum verum on Biofilm and Virulence Properties of Escherichia coli O157:H7
Source: Antibiotics (Basel). 2021 Jan 25;10(2):113. doi: 10.3390/antibiotics10020113 (PMC7911385; doi:10.3390/antibiotics10020113)
Supplement: Supplementary file 1 [file antibiotics-10-00113-s001.pdf]

Table 1S. Chemical composition of the five essential oils investigated in this study.

**Component**[a]	**RI**[b]	**RI Lit.**[c]	***C. flexuousus* (%)**	***C. citratus* (%)**	***C. martini* (%)**	***C. verum* bark (%)**	***C. verum* leaf (%)**
tricyclene	916	920	0,1	0,4			
α-thujene	920	924	tr[d]	tr	0,1	tr	0,1
α-pinene	925	932	0,1	0,4	tr	tr	0,4
camphene	938	946	0,8	1,9			0,1
benzaldehyde	954	952				tr	0,1
β-pinene	967	974					0,1
6-methyl-5-hepten-2-one	984	981	1,4	0,8			
myrcene	988	988	0,1	0,3	0,1		tr
(3*E*)-hexenyl acetate	998	1001	tr				
α-phellandrene	1002	1002				tr	0,5
δ-3-carene	1007	1008					tr
α-terpinene	1013	1015					tr
o-cymene	1019	1022					tr
p-cymene	1021	1020	tr	0,1	tr	0,1	0,7
limonene	1024	1024	0,2	0,9	0,1	tr	0,1
β-phellandrene	1024	1025					0,4
1,8-cineole	1026	1025		tr	tr	0,9	0,1
(*Z*)-β-ocimene	1036	1032	tr	0,1	0,2		
(*E*)-β-ocimene	1046	1044		tr	0,3		
pentyl propyl ketone	1071	1078	0,7	0,3			
terpinolene	1084	1086		tr			tr
linalool	1099	1095	1,0	0,7	2,1	0,1	1,1
2-methyl butyl isovalerate	1104	1103					tr
camphor	1138	1141		tr			
exo-isocitral	1144	1140		tr			
citronellal	1152	1148	0,2	0,1			
borneol	1160	1165	0,1	0,3			tr

(*Z*)-isocitral	1164	1160		0,1			
terpinen-4-ol	1171	1174		tr		0,8	tr
rosefuran epoxide	1175	1173	0,1	0,1			
(*E*)-isocitral	1182	1177		0,3			
isogeranial	1183	1184	0,3				
α-terpineol	1186	1186	0,1	0,4			0,1
cis-piperitol	1196	1195		tr			
n-decanal	1205	1201	tr	tr			
trans-piperitol	1214	1207		tr			
(*Z*)-cinnamaldehyde	1215	1217				0,2	
nerol	1227	1227		tr	0,2		
neral	1239	1235	30,0	32,0	0,1		
piperitone	1249	1249	0,1	0,1			
geraniol	1254	1249	5,5	3,1	82,2		
(*E*)-cinnamaldehyde	1267	1267				85,4	0,6
geranial	1269	1264	41,5	48,2	0,8		
citronellyl formate	1275	1271		0,2			
safrole	1283	1285				0,1	0,8
bornyl acetate	1287	1287		0,5			
geranyl formate	1301	1298	0,1	0,1	0,1		
δ-elemene	1345	1335		tr			
α-cubebene	1353	1345		0,1			
eugenol	1353	1356				7,0	83,5
α-ylangene	1353	1373	0,2				
cyclosativene	1356	1369	0,2				
(*Z*)-α-damascone	1360	1355	0,1				
neryl acetate	1363	1359	0,1		tr		
α-copaene	1366	1374	0,1	tr	tr	tr	0,9
geranyl acetate	1383	1379	6,1	3,8	11,1		
(*E*)-caryophyllene	1406	1417	1,1	1,6	2,2	0,3	2,8

(*E*)-cinnamyl acetate	1438	1443				4,5	1,2
α-humulene	1439	1452	0,1	0,1	0,1		1,2
(*E*)-isoeugenol	1442	1448		tr			
epi-cubebol	1480	1493	0,2				
γ-cadinene	1498	1513	2,6	0,9			
trans-calamenene	1510	1521	tr				
δ-cadinene	1510	1522	0,3	0,3			0,1
eugenol acetate	1520	1521				0,1	1,9
(*E*)-nerolidol	1555	1561	tr	0,2	tr		
caryophyllene oxide	1565	1583	2,8	0,4	tr		0,2
humulene epoxide II	1591	1608	0,1				
1,10-*di*-*epi*-cubenol	1599	1618		0,2			
benzyl benzoate	1752	1759			tr	0,1	2,9
squalene	2819	2819				0,3	
Total identified (%)			96,6	99,4	99,8	99,9	99,9

[a] Elution order according to a HP-5MS column (30 m x 0.25 mm, 0.1 mm f.t.). [b] Temperature-programmed linear retention index calculated using a C_8-C_{30} mixture of alkanes. [c] Retention index taken from the ADAMS library. [d] traces, % < 0.1

Fig. 1S Supplementary material. GC-MS chemical profiles of the five essential oils investigated for the anti-biofilm activity.

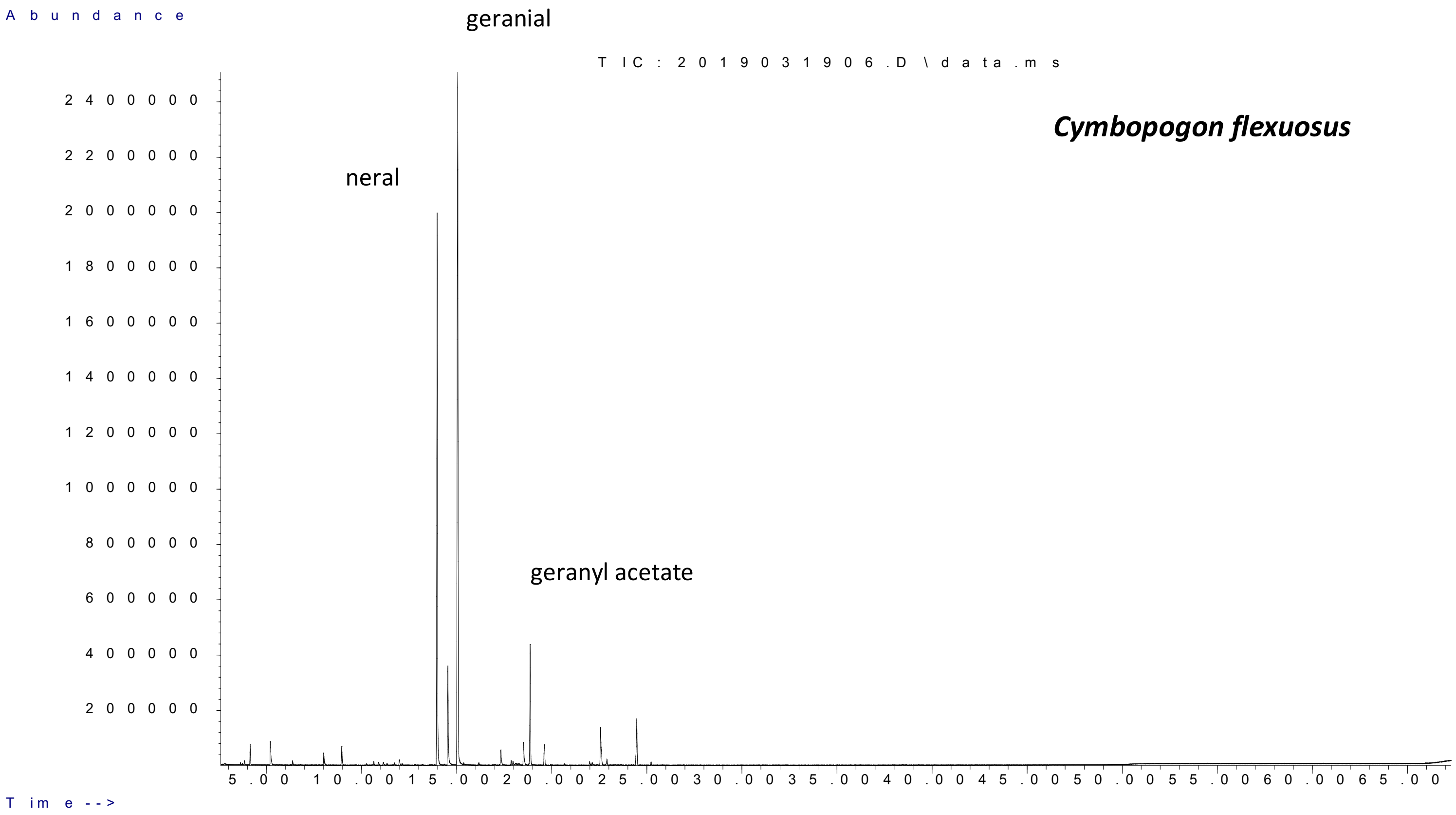

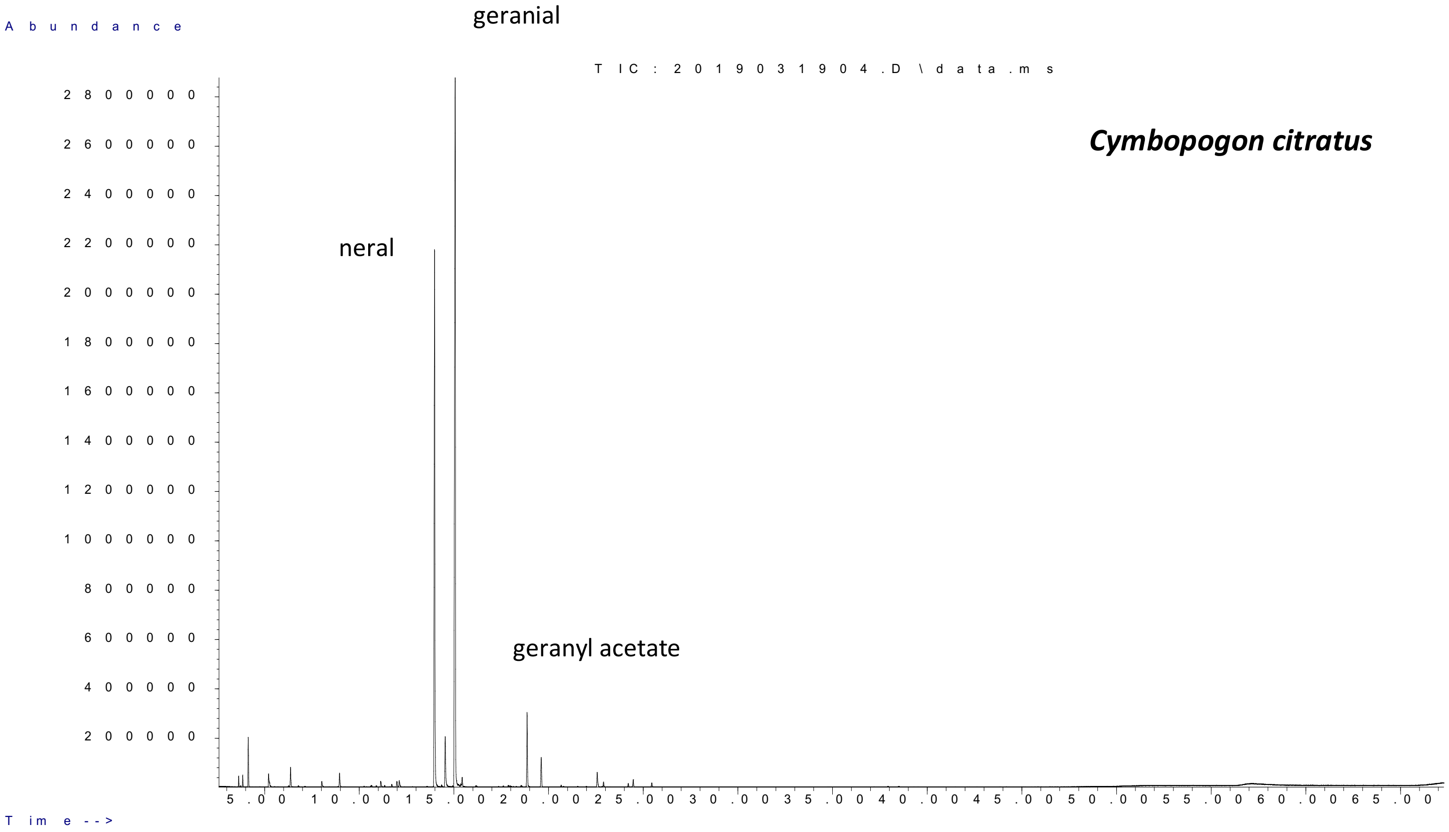

Abundance
geranial
TIC: 2019031904.D\data.ms
Cymbopogon citratus
2800000
2600000
2400000
2200000
neral
2000000
1800000
1600000
1400000
1200000
1000000
800000
600000
geranyl acetate
400000
200000
5.00
10.00
15.00
20.00
25.00
30.00
35.00
40.00
45.00
50.00
55.00
60.00
65.00
Time-->

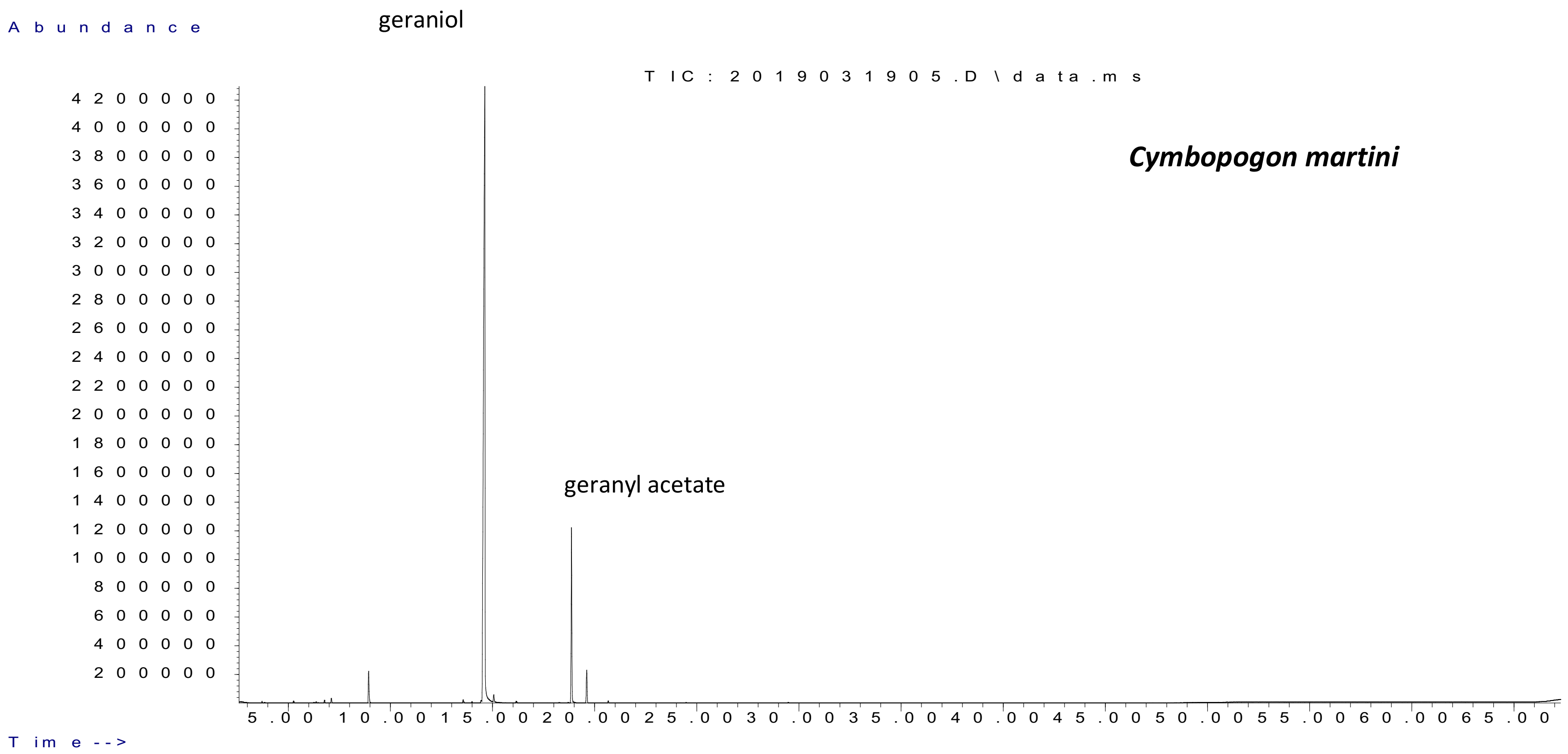
Abundance
geraniol
TIC: 2019031905.D\data.ms
Cymbopogon martini
4200000
4000000
3800000
3600000
3400000
3200000
3000000
2800000
2600000
2400000
2200000
2000000
1800000
1600000
1400000
1200000
1000000
800000
600000
400000
200000
geranyl acetate
5.00 10.00 15.00 20.00 25.00 30.00 35.00 40.00 45.00 50.00 55.00 60.00 65.00
Time-->

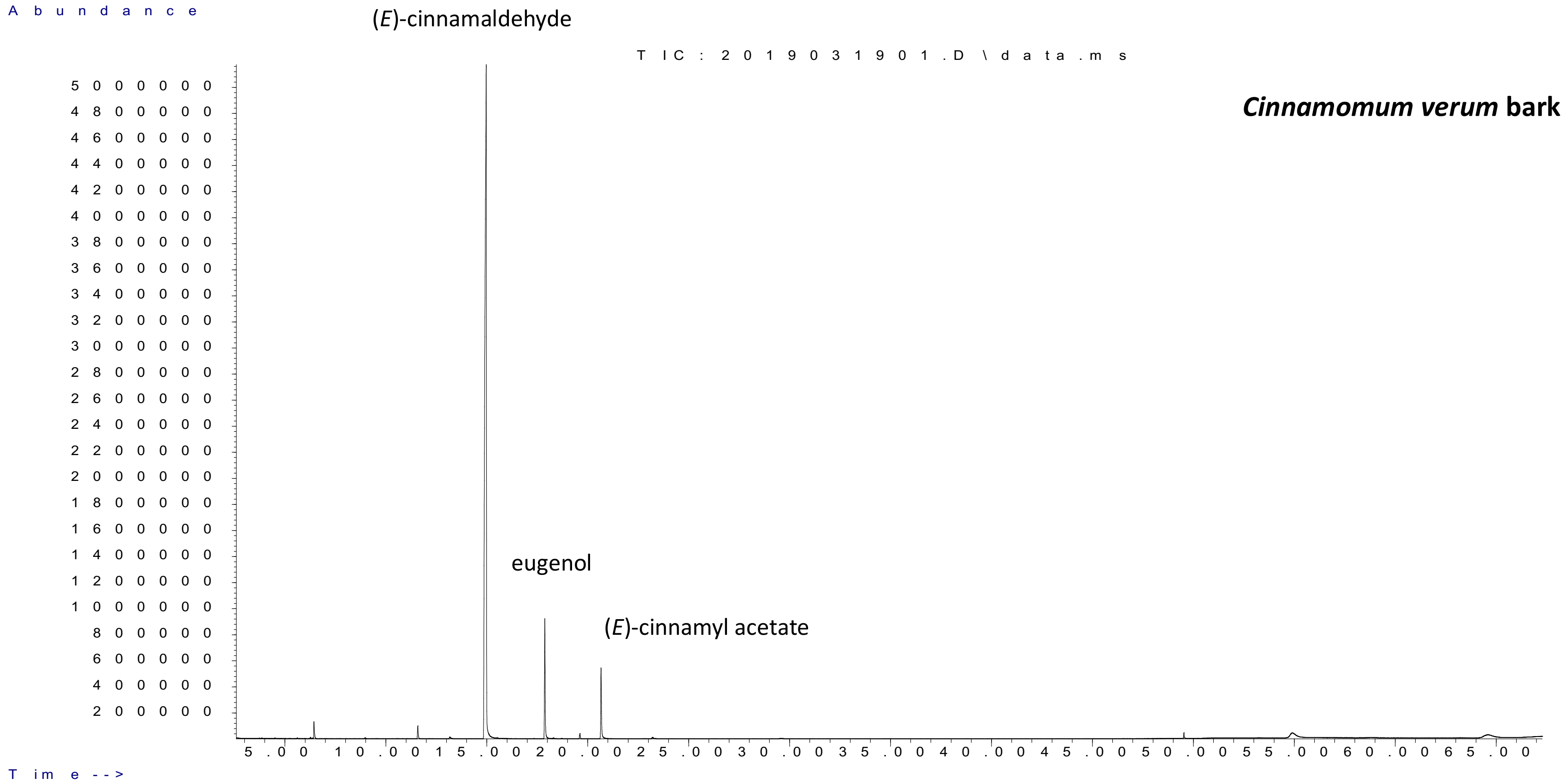

Abundance
(E)-cinnamaldehyde
TIC: 2019031901.D\data.ms
Cinnamomum verum bark
eugenol
(E)-cinnamyl acetate
Time-->

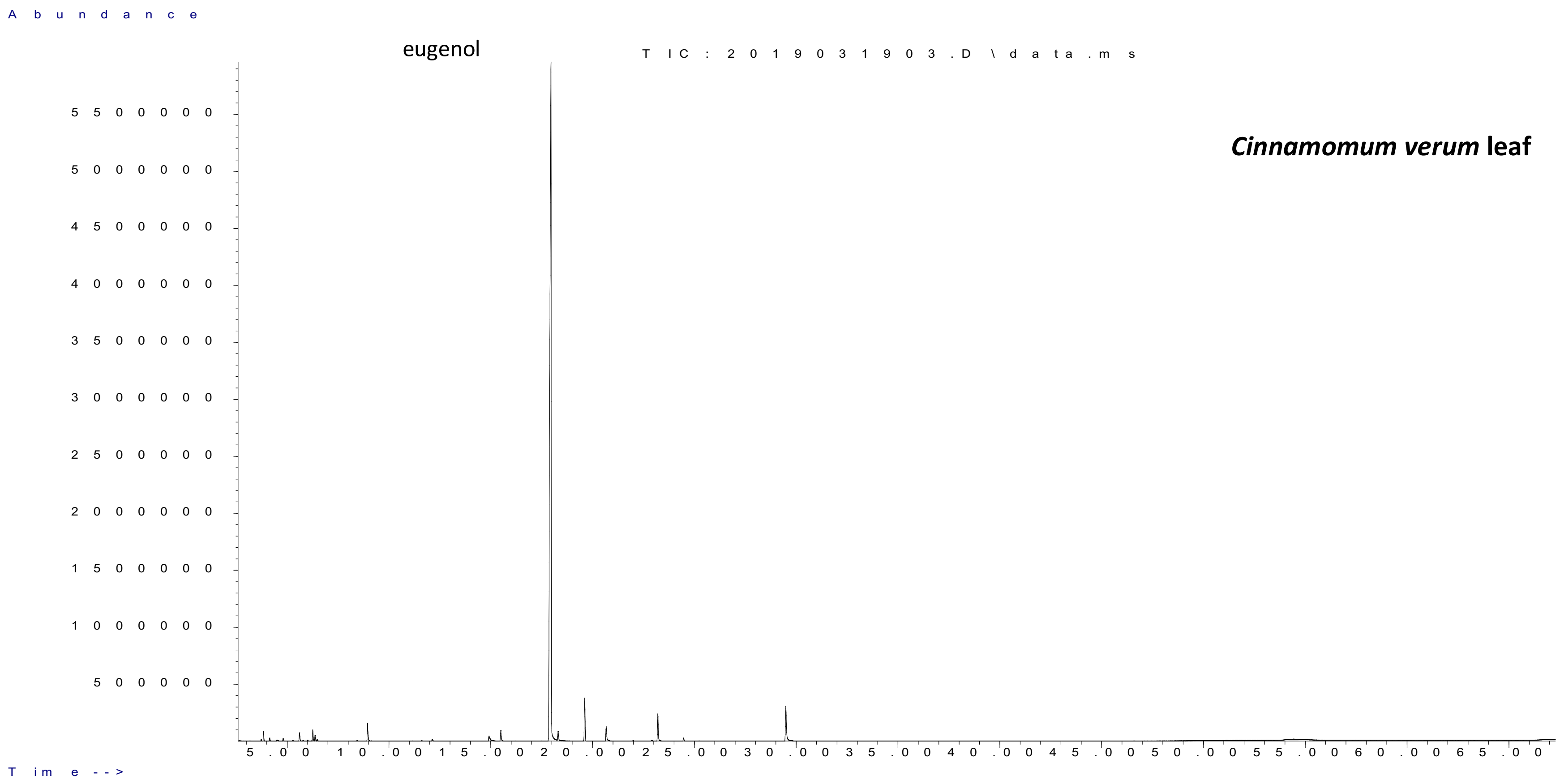
Abundance
eugenol
TIC: 2019031903.D\data.ms
Cinnamomum verum leaf
5500000
5000000
4500000
4000000
3500000
3000000
2500000
2000000
1500000
1000000
500000
5.00 10.00 15.00 20.00 25.00 30.00 35.00 40.00 45.00 50.00 55.00 60.00 65.00
Time-->
